# Individuality and temporal stability of the human gut microbiome

**DOI:** 10.5195/cajgh.2013.120

**Published:** 2014-03-27

**Authors:** Shinichi Sunagawa, Siegfried Schloissnig, Manimozhiyan Arumugam, Kristoffer Forslund, Makedonka Mitreva, Julien Tap, Ana Zhu, Alison Waller, Daniel R. Mende, Jens Roat Kultima, John Martin, Karthik Kota, Shamil R. Sunyaev, Athanasios Typas, George M. Weinstock, Peer Bork

**Affiliations:** European Molecular Biology Laboratory, Heidelberg, Germany

**Keywords:** gut microbiome, genotype, antibiotic resistance

## Abstract

**Introduction:**

The breakthrough of next generation sequencing-technologies has enabled large-scale studies of natural microbial communities and the 16S rRNA genes have been widely used as a phylogenetic marker to study community structure. However, major limitations of this approach are that neither strain-level resolution nor genomic context of microorganisms can be provided. This information, however, is crucial to answer fundamental questions about the temporal stability and distinctiveness of natural microbial communities.

**Material and methods:**

We developed a methodological framework for metagenomic single nucleotide polymorphism (SNP) variation analysis and applied it to publicly available data from 252 human fecal samples from 207 European and North American individuals. We further analyzed samples from 43 healthy subjects that were sampled at least twice over time intervals of up to one year and measured population similarities of dominant gut species.

**Results:**

We detected 10.3 million SNPs in 101 species, which nearly amounts to the number identified in more than 1,000 humans.

**Conclusion:**

The most striking result was that host-specific strains appear to be retained over long time periods. This indicates that individual-specific strains are not easily exchanged with the environment and furthermore, that an individuals appear to have a unique metagenomic genotype. This, in turn, is linked to implications for human gut physiology, such as the stability of antibiotic resistance potential.

